# On Inflammatory Affections of the Brain

**Published:** 1859-10-01

**Authors:** 


### ART. III.—ON INFLAMMATORY AFFECTIONS OF THE BRAIN.

THE physician who attempts to penetrate the pathological mysteries of the central nervous system, is liable to be led astray by three errors, or sources of error, which have hitherto infected more or less researches into the morbid conditions of the myelencephalon.

He may adopt the notion (1), that functional perversions of the nervous system are not necessarily connected with or dependent upon organic changes; or (2), that it is vain to seek for anatomical diagnostics of such cerebral or spinal affections as may be accompanied by manifest alterations in the appearance or structure of the organs, and that we must be content with marking solely the functional phenomena which may occur. In either case he assumes that the limit of his then knowledge must be the limit of subsequent knowledge; that the difficulty before which he has recoiled rests in the question to be solved, and not in the method by which the solution has been attempted; or else that that method has reached its utmost perfection, and that, consequently the question must ever remain uninterpreted—than which conceptions, whether expressed or implied, nothing could be more disheartening to the eager student, nothing more prejudicial to the advancement of science.

The third error which is apt to prove a stumbling-block to the inquirer, differs from the two former ones in character, but is of no less importance. It is (3), looseness of *nomenclature*. We have in common use, as derived from phenomena, such terms as brain-fever, acute delirium, phrensy, apoplexy, muscular paralysis, convulsions, hemiplegia, &c.; as derived from anatomical characters, hydrocephalus, atrophy, or induration of the brain, congestion, softening, encephalic hæmorrhage, &c. Of these terms the phenomenal are vague in character, and several are apt to be applied to one and the same affection; the anatomical leave us in doubt as to the nature, presumed or probable, of the organic lesions they are supposed to designate. We cannot as yet apply to all lesions of the nervous system terms scientifically precise, but we can reject terms manifestly vague and incorrect, and which are used indifferently to express several morbid conditions.

Now M. Calmeil protests against these errors, and he endeavours to clench his protest by unfolding in two portly octavo volumes* the results of thirty years' study at the bed-side and in the dead-house of one large class of cerebral affections. So long a period of research would alone claim respect for the opinions to which it has given rise, apart from the weight which must attach to the honourable and high position which M. Calmeil holds among European physicians.

M. Calmeil treats in the work referred to, (1), of sudden and transitory attacks of encephalic congestion; (2), of acute delirium; (3), of incomplete general paralysis in its simple state and (4) as complicated with other affections; (5 and 6), of acute and chronic softening of the brain; (7), of recent local encephalic hæmorrhage; and (8), of non-recent hæmorrhagic centres.

These affections M. Calmeil classes with the phlegmasia (*a*), because the causes which give rise to them are commonly recognised as being irritant, as modifying the innervation of the bloodvessels, and abnormally exciting their activity; and (*b*), because he has, as he conceives, been able to demonstrate that they are all represented anatomically, according to their phases, either by enlargements of the capillaries, by plastic effusions, or by collections of granular products, such as pus globules, pyoid globules, molecular granules, agminated cells, and sometimes by the union of all these states and of all these abnormal products. (Vol. i. p. 7.)

Consistently with this view, M. Calmeil gives as terms scientifi-

* *Traité des Maladies Inflammatoires du Cerveau; ou, Histoire Anatomo-Pathologique des Congestions Encéphaliques, du Délire Aigu, de la Paralysie Générale ou Périencephalite Chronique diffuse à l'état simple ou compliqué, du Ramollissement Cérébral, local, aigu et chronique, de l'Hœmorrhagie Cérébrale, localisé, récente ou non récente.* Par le Dr. L. F. Calmeil, Médecin-en-chef de la Maison Impériale de Charenton, Officier de l'Ordre Impérial de la Legion d'Honneur. Paris. 1859. t. 2. 8vo.

cally synonymous with those by which we have designated the affections discussed, (1), sudden encephalic fluxions of an inflammatory character; (2), insidious forms of acute periencephalitis; (3), uncomplicated, and (4), complicated diffuse chronic periencephalitis; (5), acute, and (6), chronic local encephalitis without blood-clots; and (7), recent, and (8), non-recent local encephalitis with blood-clots.

These somewhat cumbrous phrases have at least the merit of indicating clearly the author's opinions upon the pathology of the diseases to which they are applied. In fact, by applying the commonly received doctrines of *inflammation* to the post-mortem appearances, general and microscopic, of many seemingly diverse diseases of the brain, he links them together in one great class, traces them to one and the same pathological change at the foundation, and thus attempts to simplify both the theory and practice of several of the most serious diseases to which man is liable. This track has been well beaten before in regard to the particular affections included in M. Calmeil's work, but to him will in especial belong the merit of the bold generalization which we have briefly noted.

It is not an easy task to do justice to M. Calmeil's voluminous treatise, more particularly as it is ballasted by numerous and elaborate reports of cases, which in themselves alone will prove an important mine for the cerebral pathologist. We shall, however, make an attempt to show to some extent his notions upon the several affections we have already specified.

I. *Temporary sudden Encephalic Congestion.*—Cerebral congestion cannot be satisfactorily studied in itself alone. It has so many bonds of connexion with other forms of cerebral diseases, both as to causation and results, that for its right comprehension it must be considered along with, as well as apart from them. The numerous points of resemblance which exist between temporary attacks of cerebral congestion and encephalitis, strike one at the first glance.

"These two pathological states manifest themselves almost constantly under the influence of the same causes; they effect equally the sensibility, intelligence, and movement; both have their seat in the capillaries of the encephalic nervous substance; both give rise to sanguineous suffusions; both appear to be excited by a modification, in every way the same, of the normal vitality; lastly, transitory and temporary congestive states are always apt to be transformed into durable inflammatory states, whilst long-standing and moderate encephalitis is always liable to be intensely exaggerated at any moment by most violent attacks of congestion. It is difficult to conceive, then, what reasons can be advanced against the classing of temporary congestive fluxions with true encephalitis.

"Nevertheless, temporary cerebral congestions have certain traits peculiar to them. In their mode of invasion, of manifestation of the divers functional phenomena and of the sanguine turgescence which accompany them; and in the promptitude with which the species of vital erethism which determines the accumulation of blood towards the encephalon at the moment of explosion tends to decline and subside, we have excellent characteristics by which to distinguish them from other inflammatory manifestations of the intra-cranial nervous centres, and it is precisely all these considerations which have compelled us in some sort to give them a paragraph apart; but our opinion is, that henceforth one can only apply to them the name of attacks of temporary encephalitis, or attacks of temporary inflammatory cerebral congestion." (Vol. i. p. 22.)

II. *Acute Delirium.*—This affection, commonly known as *acute meningitis*, is considered by M. Calmeil to be an *insidious form of acute periencephalitis.* Under this term would be apparently included the maladies, or one or more forms of them, heretofore known as acute phrensy, acute arachnoiditis, the meningitis of adults and infants, tubercular and epidemic meningitis, acute hydrocephalus, acute general paralysis, certain varieties of eclampsia, cerebral, ataxic, and even synochal fevers.

The symptoms which would lead to the suspicion that acute insidious periencephalitis exists, vary:—

"In one form the symptoms consist especially in certain disordered conditions of the intelligence, senses, and will; the functions of the circulation, respiration, and digestion.

"Some patients are a prey to obstinate sleeplessness, turbulent petulance, and irrepressible restlessness. They are incapable of attention, speak incoherently, utter detached words, vociferate, cry out without the power of restraining themselves, and without knowing why; and strike, with the head, the elbows, or the feet, their relatives and friends. Often they think they hear noises, which are but imaginary, or see frightful forms, which do not exist; and the liquids proffered to them are hastily rejected, as if they had an offensive taste or odour. The lips are dry, the tongue glabrous, red, sometimes covered with a brownish fur; the pharynx is filled with viscid mucus; when the patient consents to drink he swallows with difficulty, or he gulps the liquid at a draught; he becomes more agitated if the gastric region be pressed; and the bowels are either constipated or relaxed. The skin is hot, dry, or moistened with perspiration; the pulse is quick, full, or small; the respiration is irregular. Almost invariably, patients of this class must be fastened to their beds.

"In a second form the general symptoms are nearly the same as those we have detailed, and the acute encephalitis manifests itself equally by lesions of the intelligence and senses; but the hallucinations assume, as also the other delirious conceptions, the characters of partial delirium. Many patients of this class have a terrified aspect; they endeavour to escape from the hands of those who have charge of them, as if their life were menaced; they expectorate around them without ceasing, as if to get rid of a suspected saliva; they oppose a vigorous resistance when it is attempted to place medicine in their mouth; they do not rest a second in the night, are assailed by menacing voices, by strange noises, and make in some instances desperate efforts to cast themselves down, or to kill themselves in some fashion.

"In a third form, the symptoms manifested by the intellectual and the sensorial functions continue the same; but these symptoms are complicated with certain abnormal states of the myotility. These lesions are scarcely appreciable, or they are very manifest. They are shown in a difficulty of speech, spasms of the facial muscles, startings of the muscles of the shoulders and arms, uncertainty of the gait, and in a graver degree, by paroxysms of epileptiform convulsions.

"Encephalitis, which takes this latter form, is rarely misconceived; nevertheless it is sometimes mistaken for a form of Saint Guy's dance, or for an attack of encephalic congestion." (Vol. i. pp. 145-6.)

The respiratory functions, and those of the alimentary canal, are frequently disturbed during the progress of acute periencephalitis, and this is important to be noted in practice. The prognosis of the disease is unfavourable. It rarely happens that the intelligence and health are completely re-established. Most commonly the affection passes into a chronic form, when it is known as *incomplete general paralysis;* occasionally it ends in simple permanent insanity.

The principal anatomical characters of the malady consist in the congested state, redness, and enlargement of the blood-vessels and capillaries, either of the cerebral pia-mater or of the cortical substance of the encephalon. To these lesions are quickly added, serous or sero-sanguinolent extravasations in the pia-mater, serous infiltration and softening of the grey matter of the turgescent convolutions, the formation either of pus-globules or of a certain number of minute punctated spherules, which seem as if they were diminutives of the large agminated cells that abound in old foci of chronic encephalitis. Acute meningo-encephalitis, although of the same nature as insidious acute periencephalitis, differs from this affection in its anatomical characters; the latter exhibiting more inflammatory congestion, but less effusion of plasma and fewer fibrinous elements.

In fatal cases of acute insidious periencephalitis, it is frequently found that the mucous membrane of the stomach and the small and large intestines, is highly congested, and that there is an inflammatory condition of the pleura and pulmonary parenchyma.

"It is," says M. Calmeil, "evidently the co-existence of these inflammatory foci which has given rise to the opinion among certain observers, that the delirium produced by the invasion of periencephalitis ought to be considered as purely a symptomatic lesion. This may be true in certain cases, but the cerebral accidents do not the less represent important material disorders, and we should reason ill to sustain that they are but the expression of a simple functional perturbation, and purely dynamic." (Vol. i. p. 149.)

III. *Incomplete General Paralysis.*—This malady, in its uncomplicated state, is best known under the term of *general paralysis of the insane*, or with mental alienation, a name originally given to the affection by M. Calmeil, but which he conceives to be now unfitted, since the true nature of the affection can be determined with some degree of certainty, it being a *diffuse chronic periencephalitis.*

This form of encephalitis has its special seat in the periphery of the cerebral hemisphere and cerebellum, but when it has endured some time, it tends to affect the deeper portions of the encephalon and the spinal cord, and sooner or later it occasions almost complete paralysis of movement, and disturbs the intelligence.

The post-mortem appearances which indicate the inflammatory character of the malady, are almost invariably readily appreciable by the naked eye. When chronic periencephalitis has lasted some time, the pia-mater is found highly congested, and there is effusion of serous, sero-fibrinous, and occasionally sanguinolent fluid into the areolar tissue which occupies the meshes of the membrane. Plastic effusions, distinguished by their opaline aspect, can also be traced along the track of the principal arterial branches. These alterations are most manifest in the interlobular fissures and the fissure of Sylvius, and they are more or less apparent according to the greater or less extension of the inflammatory action and its degree of activity.

If the pia-mater be raised from the nervous substance at a point where morbid action is marked, the congested vessels detached from the grey matter are found to form vascular and bleeding loops; and at the bottom of the principal anfractuosities the capillaries appear everywhere under the aspect of tortuous filaments. Often, however, the pia-mater cannot be detached without dragging along with it a portion of the nervous substance, which then appears excoriated, torn, red, and bleeding, more or less softened, indurated, or atrophied.

It is difficult to describe the aspect of those regions to which the pia-mater adheres intimately in the gravest cases of chronic encephalitis; but it may be said that the exposed surfaces of the brain and cerebellum have an ulcerated and ragged appearance, and that they are covered with nipples of greater or less prominence and depressions of different depths. In some instances the tearing of the grey substance is very slight and superficial, and occurs only in spots of slight extent, which might without care escape the uninstructed eye, but the structural lesions at these points differ only in degree from the more manifest changes alluded to.

The nervous substance surrounding and upon the borders of the apparent ulcerations will be found softened to a greater or less extent if it be examined with the edge of the scalpel. This diminution of consistence may extend deeply into the cortical layer; and it sometimes happens that while the central portions of this are softened, the superficial portion is hard and brittle.

When the substance of the convolutions is cut into, it is found to have a red, violet, or reddish tint, due to the accumulation of blood in the capillaries, or to changes undergone by extravasated hæmatosine. At times numerous specks of blood escape from the divided bloodvessels, and mark their state of injection.

Certain portions of the cerebral hemispheres are more affected by chronic diffuse encephalitis than others. The parts most commonly occupied by morbid changes are the convolutions bordering the fissure of Sylvius, those right and left of the falx cerebri, the convolutions which correspond to the inferior part of the anterior lobes, and the superior, lateral, and convex regions of the posterior and middle lobes. The superior and inferior faces of the cerebellum are the portions of that organ chiefly affected.

In general the inflammation extends only a few millimetres into the thickness of that portion of the nervous substance which is in immediate relation with the meninges, but when the inflammatory action passes beyond a certain degree of intensity, the grey matter of the corpora striata, the optic thalami, and the cornua Ammonis, is often affected.

When the white substance of the cerebral hemispheres and the cerebellum is cut into, it is found sanded, as it were, with bloody spots.

All these appearances point to long-continued inflammatory action, and microscopic observation of the affected tissues leads to the same conclusion.

Under the microscope, the vessels of the pia-mater are seen to be tortuous, red, and congested; while in the meshes of the tissue, and the effused serum occupying them, are found extravasated blood-globules, and a greater or less amount of granular cells and molecular granules. Pus globules are also occasionally met with. The capillaries of the pia-mater are frequently powdered, as it were, with a thick coating of fine molecular granules, which incrust them as a bark.

Many times portions of grey substance, as yet not softened, and from the centres of morbid action, have appeared, under a magnifying power of from 400 to 450 diameters, as if furrowed by considerable vascular arborisations. Many of these vessels divided and subdivided, and ended by forming a species of plexus. Many of them also contained a column of liquid blood, others contained but a mass of heaped-up blood-globules, but all were noted by the enlargement of their calibre. Many capillaries were encrusted exteriorly with molecular granules, either whitish or blackish; others were as if strewed with small agminated cells, which abounded in the bifurcations formed by the branching of several vascular trunks. (Vol. i. p. 266.)

Where there was an evident defect in the consistency of the grey substance, on handling it in order to prepare it for examination under the microscope, a liquid was expressed, which contained blood-globules and the varieties of cells already described, but no disintegrated portions of nervous tissue. When the cortical substance was decidedly softened, the liquid which penetrated it was augmented in abundance; the nervous tissue was for the most part disintegrated and reduced into little particles of a greyish colour, and mingled with altered blood-globules; agminated cells were visible everywhere, but always small, and composed of very fine granules.

The foregoing morbid appearances, as well general as microscopical, according to M. Calmeil, differ in no respect from those we are accustomed to find in the majority of inflamed parts, and therefore the disease which gives rise to incomplete general paralysis, with lesion of intelligence, merits well the name of periencephalitis. Nevertheless, it is to be noted that "diffuse chronic periencephalitis of little intensity, and which is manifestly uncomplicated, partakes much more of chronic inflammatory congestion, accompanied by serous or sero-fibrinous extravasation, poor in fibrine, than of congestion with extravasation of a notable quantity of fibrinous plasma."

It must be added, that the vessels which ramify on the surface of the great ventricles and the capillaries of the grey substance of the corpora striata, optic thalami, cornua Ammonis, and the annular protuberance, exhibit more or less turgescence in individuals affected by diffuse chronic periencephalitis. We are, then, justified in believing that the inflammatory action tends to affect these different localities.

The vessels of the white substance, also, under a lens of slight power, are seen to be of greater capacity than is customary, and occasionally they are found to be covered with a species of granular incrustation.

We cannot follow M. Calmeil at length in his elaborate account of the symptoms of diffuse chronic periencephalitis, but his description of the precursory and initiatory symptoms is too valuable to be passed over :—

"In a very great number of subjects, the definitive outbreak of diffuse chronic periencephalitis is preceded by a period of remarkable functional aberrations which it is difficult to overlook. During this species of inflammatory incubation, it often happens that the moral and intellectual characteristics of the patient become completely changed. Sadness is replaced by gaiety which tends to extravagance; diffidence gives place to an assurance which is manifested in the gait, in the discourse, and which sometimes degenerates into a petulance of action and an exuberance of language fatiguing to those around. Some patients keep a-foot during the greatest part of the night, speaking, composing, writing, or constantly moving about, without undergoing any lassitude, whilst all around them are overcome with fatigue and sleep; others are irritable, apt to anger, vain, eager for novel emotions; others neglect their duties, their interests, in order to enter into speculations which the most ordinary good sense condemns, but which to them seem proper to double or quintuple their fortune; others become incapable of attention, forget those things which they knew best, hastening rapidly towards a complete fatuity; often, also, a commencing impediment in the pronunciation, mingled with a want of harmony and certainty in the movements, characterizes this period, of which the phenomena are susceptible of numerous other variations.

"In many cases, on the contrary, the invasion of diffuse chronic encephalitis is not first announced by any perturbation in the intellectual functions, but its first symptoms are manifested as the sequel of a comatose attack, caused by the sudden determination of an extraordinary quantity of blood towards the capillaries of the encephalon. . . . . .

"At the incontestable outset of this inflammation, those who have the pronunciation first affected manifest the impediment of speech most strikingly when they are intimidated or excited. Now and then, their lips are agitated by a species of undulatory startings when they open the mouth in order to express an idea, and then they pronounce imperfectly the final words which they seek to articulate well, and their tongue, when protruded, exhibits a vacillating movement; but these first symptoms are not always equally apparent at different hours of the day.

"Almost invariably, at the commencement of this phase of the inflammation, the muscles of the limbs and trunk suffer also from the effects of the changes going on in the periphery of the encephalon, and the following symptoms are noted :—The gait of the patients tends to become uncertain and irregular, their pace appears constrained, the movements of their arms imperfectly co-ordinated. The majority of them, nevertheless, continue to move about and attend to business, make visits, and go on foot, as if they had perfect health.

"Except in a few cases, lesions of sensibility are not easily demonstrated in this period of the disease; enfeeblement or loss of sight, either on one side only or on both sides, coincides, nevertheless, sometimes with the manifestation of the first symptoms of impediment of speech; the tactile sensibility is probably blunted, also, at this epoch, because the majority of the paralytic insane appear to be hardly conscious of their hurts.

"There is almost invariably lesion of the intellectual functions when diffuse chronic periencephalitis has acquired sufficient intensity to affect evidently the exercise of voluntary movements. All patients do not necessarily become delirious, all do not necessarily lose then their habitual judgment; but if they be submitted to an attentive examination, it will be discovered, nearly constantly, that there are, even when the inflammation is but slightly advanced, either signs of delirium, or irrational conceptions, or symptoms of commencing, or even already very manifest dementia.

"Maniacal petulance, exaggeration of ambitious ideas, predominance of a certain number of melancholic ideas, and powerlessness of the intellect, constitute the types of mental alienation or intellectual enfeeblement which require to be particularly noted in the first stage of diffuse chronic periencephalitis. . . . . . . . .

"The predominance of ambitious conceptions is most frequent in those individuals whose encephalic nervous centres are menaced, and, indeed, are already attacked by chronic and progressive diffuse inflammation. At first, the subjects who are suffering from this malady exhibit a certain degree of reserve in speaking of their dignities, their titles, their acquisitions, their riches, and the elevation which they expect, and they consider twice before they proclaim publicly and haughtily that they will presently be seated upon some throne. They fear still to encounter incredulity in endeavouring to pass themselves as illustrious conquerors, and in claiming those honours which are the apanage of great fortunes; but quickly all hesitation terminates, and they assert with a kind of joyous emphasis before every one that they have discovered mines of gold, that they possess mines of diamonds, that they are about to erect sumptuous palaces, that they surpass the greatest painters and poets in talent, that they can resuscitate the dead, create anew the world, and dismay armies by the force of their will. All these follies are uttered with quiverings of the voice which leave no doubt as to the impediment of the organs of speech, and they are accompanied with demonstrations of joy, satisfaction, and contentment, which form a marked contrast with the painful impression which they never fail to produce upon those before whom the delusions are manifested.

"The melancholic type occupies also an important place among the functional manifestations of diffuse chronic periencephalitis, and for a period of ten years this form of delirium has been manifested, in subjects suffering from the initiatory stage of general paralysis, nearly as frequently as ambitious monomania. Thus, among the individuals of whom we purpose to speak presently, there are found solely discouraging, fearful, and horrible ideas. Some imagine that they are about to be guillotined, others that they are calumniated, others that it is sought to poison them; all, or nearly all, have a wretched appearance and countenance, they refuse to speak, to do anything, to take their food, and the efforts made in order to make them eat and to feed them are rarely followed with success, so that these paralytic lypemaniacs succumb generally much more rapidly than melancholics not paralysed.

"Hallucinations, more or less active and variable in their form, and affecting the sight, the hearing, and the visceral sensibility, are often added, among paralytics suffering from the beginning of diffuse cerebral inflammation, to the symptoms of mania, of ambitious monomania, and lypemania; but, in general, the hallucinations tend to disappear in proportion as the inflammatory action tends to induce disorganization of the cortical nervous element.

"Enfeeblement of memory and obliteration of the understanding, complicated or not with delirium, and with or without appearance of irrationality, ought to be accounted among the most insidious and most ordinary manifestations of commencing chronic periencephalitis. The importance of these phenomena cannot remain long misunderstood when the patients are the first to remark that they often forget dates and make omissions which they seek to avoid; when they complain of a failure of attention, either when they listen, or when they read, or when they write; when they become confused in their calculations and affirm that they have not the capacity necessary to superintend their domestic interests, to fulfil the obligations attached to their responsibilities, and to conduct properly their commercial and industrial undertakings.

"When these first signs of dementia are mingled with absurd conceptions, ideas of grandeur and opulence, frequent ravings, symptoms of exaltation, and sleeplessness; and the patients prove by the character of their actions that they no longer exercise any control over their decisions, it is still more easily and immediately perceived that the encephalic inflammation has already gravely compromised the organ of thought.

"It is easy, on the contrary, to be deceived with regard to the true state of the organs which serve for the manifestations of the understanding, when the subjects who manifest the first muscular symptoms of incomplete general paralysis in nowise fall from their accustomed tone of thought and feeling, when they do not cease to give proof of perfect rectitude and judgment, and they continue to manifest the bearing and conduct of sane men.

"Experience, nevertheless, has convinced us that these appearances ought to inspire only dubious confidence, because they serve nearly always to mask the invasion of dementia. When patients are examined with care who have at the time a difficulty in articulation and in governing their general movements, we may quickly assure ourselves, nearly always, that the operations of their understanding are less facile and prompt than was formerly the case, that their conversation has become barren, that they devote much time to make and correct their letters and in collecting their ideas, that they are irresolute, hesitate to begin anything, and have little confidence in themselves. On the other hand, the friends, the relations who have familiar intercourse with them, will tell you that they repeat without knowing it, that their principles are not so elevated as they have been, and that the field of their conceptions becomes narrower and narrower. They are then upon the brink of dementia.

"I do not affirm that chronic periencephalitis always and necessarily (*qu'il soit dans l'essence*) affects the intellectual faculties, but I do not hesitate to affirm that it very rarely spares them. (Vol. i. pp. 274-278.)

We cannot follow further in detail M. Calmeil's carefully wrought description. But we would add, from his summary, that incomplete general paralysis especially attacks males, young, robust, sanguine, and with a well-developed muscular system; it is apt to be induced by all causes which exercise an irritating effect upon the nervous system; after some months' duration (twelve or fifteen) it often causes a general powerlessness of all the muscles, and, more or less, complete abolition of the intellectual functions; it often manifestly affects the senses and the transmission of tactile and visceral impressions; it is occasionally aggravated suddenly by intercurrent congestive attacks, and its progress is at times interrupted by remissions; it is essentially grave in character, but its degree of gravity depends in part upon the extent of surface which it affects or the depth to which it penetrates in the encephalon.

Is incomplete general paralysis a hopeless disease? When the malady is fully formed, the weight of authority is in the affirmative. M. Calmeil's opinion is very far from encouraging.

"Physicians who have observed but doubtful cases, or but few instances of diffuse chronic periencephalitis easily confound the remissions of this affection with cases of cure; but those who have continued their observations over a greater period than a year, and who have studied the course of periencephalitis in great hospitals, are nearly unanimous in proclaiming the rarity of true cures. We ought, then, to feel glad if after many able combinations we have succeeded in retarding notably the progress of the disease, or in bringing about intermissions of some duration." (Vol. i. p. 286.)

The chief points in the treatment of the disease are as follows:—

Removal of the patient to an asylum, or to a commodious house in the country, where he may be entirely separated from the influence of business or family cares; a generous, but not rich or stimulating diet; diluent and saline drinks; if young and robust small general and local bleedings, which are to be repeated at intervals, according to the effects produced upon the symptoms—ordinarily at intervals of one or two months; warm baths, prolonged during three, four, or five hours, with the application of cold to the head or the douche, according to the degree of maniacal petulance or of fury; hot pediluvia with sinapisms, or pediluvia with chlorhydric acid, and purgatives.

The activity of antiphlogistic measures must be diminished if the loss of memory, the obliteration of the mental faculties, and the difficulty of speech augment, notwithstanding the remedies used, and recourse must be had to setons, blisters, or cauterization.

So soon as indications of serous infiltration, or softening and disintegration of the brain, become evident, all curative treatment should be renounced. The physician's duty is then perforce confined to directing such hygienic care as may best conduce to the comfort of the patient, except when furious exaltation supervenes as a sequel of intercurrent congestion, which must be met by such a combination of the measures already mentioned as experience in each case alone can determine.

Diffuse chronic periencephalitis does not always run a simple course. Often it is complicated by alarming apoplectic symptoms, coma, paroxysms of convulsion, or by the paralysis tending to manifest itself more markedly in certain of the limbs. These phenomena are to be attributed to the supervention of inflammatory and congestive recrudescences; the course of the chronic inflammation is, in fact, traversed by true accessions of acute encephalitis. These recrudescences may be localized in any portion of the encephalon, and their seat after death is readily distinguished by the usual products and effects of inflammation. The complications must be combated by a well-regulated antiphlogistic and hygienic treatment.

IV. *Softening of the Brain.*—M. Calmeil treats of *acute* and *chronic* cerebral ramollissement, the former being distinguished as acute, the latter as chronic *local encephalitis without blood-clots,* seated under the form of circumscribed foci, either on the surface, or in the depths of the encephalon.

*Acute local encephalitis without clot* is brought about by the same mechanism as the great cerebral hæmorrhages, but it differs from the latter in this respect, that the congested capillaries pour into the interstices of the nervous tissue plasma and a few blood globules, but not an enormous quantity of blood itself. The study, then, of this form of encephalitis will include the so-called sanguineous infiltrations, ramollissements *of every colour,* recent abscesses, whether encysted or not, these as well as other cerebral lesions, originating in, and being consequences of, localized inflammatory action.

The changes manifested in the inflamed cerebral tissue in different stages of local encephalitis may be summed up thus :—

(1.) Congestion and redness of the capillaries. (2.) *Hepatisation* of the inflamed nervous substance. (3.) *Softening.* During the period of *hepatisation,* the inflamed centre presents, under the microscope, besides repletion of the capillaries, extravasated and granular elements; during the period of *softening* the nervous substance is found to be permeated with serum and fibrinous exudation, softened, stuffed as it were, with spherical granules, large granular cells, and occasionally pus globules; and the nervous tissue itself is found to be more or less broken down. True *abscess* of the brain differs but little from ordinary foci of *ramollissement,* the number of pus globules in the former being much more considerable than the number of large granular cells within their detritus.

Acute local encephalitis may occur in one spot only of the cerebrum or cerebellum, or it may have many seats, either on one or both sides of the encephalon. The morbid action may not occupy a space larger than a pellet, or it may equal in volume an almond or an apple, or it may even extend to nearly the whole of one hemisphere.

Chronic local encephalitis differs from the acute in the duration or intensity of the inflammatory action, and after death, cysts filled with pus, granules, agminated cells, or a melange of these matters, cicatrices, or sundry membraniform structures, formed from the plastic fluids effused into the inflamed parts, are often met with.

V. *Cerebral hæmorrhage.*—Sanguineous apoplexy, interstitial cerebral hæmorrhage, according to M. Calmeil, is occasioned by the same causes, and is developed under the same circumstances, as every form of encephalitis. It ought, therefore, to be considered as an acute local encephalitis with extravasation of blood. It resembles in almost every particular acute local encephalitis without hæmorrhage, or local cerebral ramollissement, differing from this affection chiefly in the extravasation of blood. It may occur at a point already softened and affected with local acute encephalitis, or it may take place in a spot free from all inflammatory action. In effusions of the first character which prove suddenly fatal, the granular products of inflammation are found in abundance in the softened nervous tissue, but not in the recent coagulum; in those effusions which take place in a locality free from all anterior inflammatory action, and during the commencement of the period of congestion, there does not exist at the seat of rupture, neither in the torn tissue nor in the blood, any granular element. Hæmorrhagic foci, seven or eight days old, resemble in almost every respect foci of ramollissement without clot; they differ little except in the volume of the coagulum contained in them. It is not necessary in order that a hæmorrhagic focus should form in the cerebrum, that the nervous substance should invariably have been previously softened, but it is occasionally affected at the moment of effusion by softening more or less manifest. The diminution of consistency in the nervous substance which is found immediately after the formation of an effusion during the period of congestion, depends solely upon the presence of effused serous and fibrinous liquids, because no granular cells are present, and the tissue is not much disintegrated; but the softening which supervenes a few days after the formation of the coagulum is characterized by an abundance of granular cells and the complete disintegration of the nervous fibres.

On the changes which occur in the seats of cerebral hæmorrhage, when this does not quickly compromise life, or of the *non-acute stage of local encephalitis* with extravasation of blood, we shall not dwell, although these changes and the symptoms they give rise to are discussed at length by M. Calmeil.

Our main object has been to convey some notion of M. Calmeil's pathological opinions, for these constitute the most novel portion of his work—we will not say most interesting, or most valuable, since this would be a moot point with many persons. For there are some who will doubtless regard M. Calmeil's admirable account of the symptoms and progress of the affections of which he treats as of greatest value; others the numerous and careful reports of cases and summaries of facts which he gives. Be this as it may, M. Calmeil's pathology has an immediate and important bearing upon his practice at the bed-side,—it in fact governs that practice entirely; nay more, it is requisite to know M. Calmeil's practice, in order fully to appreciate his pathology. He discusses the treatment proper to each of the cerebral affections of which he writes in its proper place apart, and at the termination of his work he considers at length the treatment proper to be pursued in inflammatory affections of the brain.

Let it be premised here that the causes which predispose to, or determine idiopathic cerebral affections, are the same under every variety of form of those affections. Hereditary predisposition plays an important causative part in many instances; men with voluminous hearts, well developed muscular systems, full blooded, and of hasty, overbearing, passionate character, are liable to these maladies. Males are more liable than females, but the liabilities to the different forms of inflammatory cerebral diseases, differ at various periods of life. Powerful emotions of every kind are apt to determine attacks; so also and very markedly the imbibition of spirituous liquors. Active exercise under a hot sun and certain not well-defined meteorological changes, are also influential causes.

A knowledge of these causes forms the basis of a well-considered preservative treatment, and M. Calmeil particularly insists on the importance of such a treatment in the case of children whose parents and ancestors have shown a marked predisposition to head affections, and who themselves have suffered from any cerebral disturbance in infancy, or manifested precocity in childhood. Such children should be pursued by a carefully regulated hygiene, particularly in their school days, and much judgment should be exercised in the selection of a profession for them. It is not necessary, however, to enter into particulars respecting the methods of care which should be had recourse to; these can hardly be overlooked as soon as the predisposing and exciting causes of the diseases are rightly apprehended.

The treatment to be adopted during the initiatory stage and culmination of encephalic inflammation, should be, according to M. Calmeil, essentially antiphlogistic. His sheet-anchor is the lancet. Full and reiterated bleedings from the arm, according to the effects produced upon the symptoms, and the age, and general condition of the patient; while, when the lancet may be inadvisable, or, as a concomitant of it, the cupping-glass and leeches are to be freely used. Blood-letting in fact, general and local, is the great remedy, and its use is to be governed by those rules which are set forth in every work on practical medicine. Along with blood-letting purgatives may be used, and the rigid and careful application of cold (particularly ice) to the head, vesicatories, with diluent and saline drinks, complete the antiphlogistic scheme requisite in cerebral affections characterized by inflammation. Compression of the carotids may be used with benefit during convulsive paroxysms. The diet, of course, must be simple, plain, and non-stimulating, and steadily watched.

In the chronic state of these affections carefully regulated blood-letting, general or local, must be made use of either to combat the progress of the affection, or to check or overcome recrudescences, together with purgatives, frequent use of the warm bath, immersing the patient three, four, or even five hours, cold being applied to the head at the time; vesicatories, setons, and moxas, and a carefully regulated diet.

Now the treatment recommended by M. Calmeil clearly indicates (what, however, is very apparent from the general tendency of his reasoning) the vice of his whole book. He speaks of inflammation as if it were a clearly defined pathological state, subject only to variations in degree of intensity, and consequently to be combated invariably by the same means applied with different degrees of vigour. He does not seem to recognise, or he lays slight stress upon, the modifications which inflammatory action undergoes from an impoverished or vitiated state of the blood, or of the system generally, and that the indications of treatment differ much, under these circumstances, from those applicable to inflammatory affections of a sthenic character. He, in fact, governs his treatment of so-called inflammatory affections of the brain by a preconceived, but limited idea of the nature of inflammatory action, and he does not appear to us to recognise sufficiently the extent to which the character of the morbid change may be affected by a co-existent abnormal condition of the system. Yet it can hardly be questioned that such a condition, whether in the form of Bright's disease, scrofulous taint, febrile infection, or anæmia, modifies very considerably the progress, results, and general character of inflammatory cerebral affections, and that it should exercise a most important influence upon the nature of the treatment adopted. Upon this question, so clearly recognised by English physicians, we need not dwell, otherwise than to point out that it is not sufficiently considered by M. Calmeil.

Notwithstanding, however, this, to us, serious drawback, we would say, in conclusion, that M. Calmeil's treatise, from the vast amount of valuable matter which it contains, and from the distinguished character of the writer, claims a very honourable position in medical literature, and it will form a fitting companion to Lallemand's anatomico-pathological researches on the encephalon.

---

